# Retraction Note to: miR-320a affects spinal cord edema through negatively regulating aquaporin-1 of blood–spinal cord barrier during bimodal stage after ischemia reperfusion injury in rats

**DOI:** 10.1186/s12868-021-00669-6

**Published:** 2021-11-03

**Authors:** Xiao-Qian Li, Bo Fang, Wen-Fei Tan, Zhi-Lin Wang, Xi-Jia Sun, Zai-Li Zhang, Hong Ma

**Affiliations:** Department of Anesthesiology, First Affiliated Hospital, China Medical University, Shenyang, 110001 Liaoning China

## Retraction Note: BMC Neurosci (2016) 17:10 http://doi.org/10.1186/s12868-016-0243-1

The Editor has retracted this publication because of significant concerns regarding a number of Figures presented in this work, which question the integrity of the data. Specifically,

- Figure 2b, it appears the same feature of staining appears in both panels for GFAP in IR group at 12 and 48 h.
- Figure 5C also appears to have some similarities between images with different labels and from different time points.

The authors were unable to provide a satisfactory explanation for these. In addition, further investigation found irregularities in some of the Western blots provided in the article. The authors were unable to provide raw data to support all the Figures.

The Editor therefore no longer has confidence in the integrity of the data in this article.

Author Xiao-Qian Li stated on behalf of all co-authors that they agree to the retraction, but not to the wording of this retraction notice.

